# Functional expression of human prostaglandin E2 receptor 4 (EP4) in *E. coli* and characterization of the binding property of EP4 with G_α_ proteins

**DOI:** 10.1016/j.bbrep.2020.100871

**Published:** 2020-12-16

**Authors:** Nam Hyuk Kim, Key-Sun Kim, Sang Chul Shin, Eunice Eunkyeong Kim, Yeon Gyu Yu

**Affiliations:** a Department of Chemistry, Kookmin University, 77, Jeongneung-ro, Seongbuk-gu, Seoul, 02707, Republic of Korea; b Convergence research Center for Diagnosis Treatment and Care System of Dementia, Korea Institute of Science and Technology, Republic of Korea; c Biomedical Research Institute, Korea Institute of Science and Technology, Seoul, 02790, Republic of Korea

**Keywords:** EP4, GPCR, PGE2, Overexpression, Purification, G protein

## Abstract

Human prostaglandin E2 receptor 4 (EP4) is one of the four subtypes of prostaglandin E_2_ (PGE_2_) receptors and belongs to the rhodopsin-type G protein-coupled receptor (GPCR) family. Particularly, EP4 is expressed in various cancer cells and is involved in cancer-cell proliferation by a G protein signaling cascade. To prepare an active form of EP4 for biochemical characterization and pharmaceutical application, this study designed a recombinant protein comprising human EP4 fused to the P9 protein (a major envelope protein of phi6 phage) and overexpressed the P9-EP4 fusion protein in the membrane fraction of *E. coli.* The solubilized P9-EP4 with sarkosyl (a strong anionic detergent) was purified by affinity chromatography. The purified protein was stabilized with amphiphilic polymers derived from poly-γ-glutamate. The polymer-stabilized P9-EP4 showed specific interaction with the alpha subunits of G_s_ or G_i_ proteins, and a high content of α-helical structure by a circular dichroism spectroscopy. Furthermore, the polymer-stabilized P9-EP4 showed strong heat resistance compared with P9-EP4 in detergents. The functional preparation of EP4 and its stabilization with amphiphilic polymers could facilitate both the biochemical characterization and pharmacological applications targeting EP4.

## Highlights

• Prostaglandin E2 receptor 4 (EP4) was overexpressed as P9-fusion protein in *E. coli*.
• The APG-stabilized P9-EP4 showed specific interaction with the alpha subunits and its ligands.
• The APG-stabilized P9-EP4 showed strong heat resistance compared with P9-EP4 in detergents.
• The binding kinetics of P9-EP4 with both antagonists and agonists were analyzed.

## 1 Introduction

Prostaglandins have multiple roles in human physiological processes and are synthesized from membrane-derived arachidonic acid via the reactions of cyclooxygenases and prostanoid synthases [1,2]. The five major prostaglandins referred to as prostanoids are prostaglandin E2 (PGE2), PGI2, PGD2, PGF2, and thromboxanes. PGE2 is synthesized from prostaglandin H2 (PGH2) by prostaglandin E synthases and regulates multiple physiological processes, including inflammation [[3], [4], [5]], reproduction [6], and tumorigenesis [7,8]. PGE2 activates four different prostanoid receptors (EP1, EP2, EP3, and EP4), which are in the superfamily of G protein-coupled receptors (GPCR). These receptors originate from four distinct genes and are distributed in different tissue or cell types [9]. The EP1 receptor activates PLC and increases in intracellular Ca^2+^ levels via coupling to G_q_ [10], while the EP2 receptor couples to G_s_ proteins, increases cAMP levels [11] and activates PKA. The EP3 receptor exists as multiple alternative splice variants resulting in different forms at the C-terminal tail region; these forms can couple with various G proteins, including G_s_, G_i_, or G_13_ [12].

The EP4 receptor is expressed in various tissue cells, including immune, cardiovascular, gastrointestinal, respiratory, and various cancer cells [13]. It couples with G_s_ proteins and activates PKA, thereby increasing levels of intracellular cAMP. EP4 also couples with G_i_ and activates PI3K and Akt [14]. Gene-knockout experiments confirmed that EP4 is involved in inflammatory and pain responses and suggest that EP4 is a promising drug target against chronic inflammatory diseases such as rheumatism [15]. The EP4 receptor is also implicated in the development of breast, prostate, and colon cancers [13]. The knock down or antagonist of EP4 mitigates the progression of various cancers; therefore, a few efficient EP4 antagonists have been tested for use in cancer treatment.

Recently, an EP4 crystal structure was reported by Yosuke Toyoda et al. [16]. A complex structure of Fab and the mutant form of EP4 (in which the intracellular loop has been deleted) was crystallized along with an antagonist. Using this structural information, the ligand binding site was estimated to be near the antagonist binding region that was surrounded by the 5th, 6th, and 7th transmembrane helical regions of EP4. Although, the crystal structure revealed detailed information about the antagonist binding site, structural information on the interaction site of EP4 with the G protein is limited. This is because the EP4 protein used for the structural analysis lacks the critical intracellular loop region that is involved in the G protein interaction. Hence, a purified active form of EP4 is required for the biochemical analysis of EP4, including its interaction with G proteins as well as chemical regulators. Furthermore, an active form of EP4 could facilitate the development of antibodies for therapeutic application.

We previously established an expression system of human GPCR in *E. coli* with an N-terminal fusion of the P9 envelope protein of Pseudomonas Phi6. Lysophosphatidic acid receptors (LPA_1_ and LPA_2_) and the endothelin receptor (ET_A_) were successfully expressed in the membrane fraction of *E. coli* as P9 fusion proteins; the purified proteins were functionally active forms [[17], [18], [19]]. The P9 domain significantly enhance the expression level of the fused GPCRs in the membrane fraction of *E. coli* instead of aggregated in the cytoplasmic fraction [19]. The P9-assisted expression system of GPCRs would facilitate the purification and biochemical characterization of GPCRs. This study prepared a C-terminal deleted form of EP4 as a P9-fusion protein in *E. coli*. The expressed P9-EP4 was successfully purified, and the purified proteins showed specific binding both with G_αi_ and G_αs_. The binding kinetics of P9-EP4 with both antagonists and agonists were analyzed by the spectral change in tryptophan (Trp) fluorescence. These results could facilitate the biophysical analysis of EP4 and the development of anti-EP4 drugs.

## 2 Materials and methods

### 2.1 Materials

The cDNA of EP4 was obtained from OriGene (USA). PGE2, isopropyl-β-D-thiogalactoside (IPTG), phenylmethylsulfonylfluoride (PMSF), and sarkosyl were obtained from Sigma (USA). The polyclonal P9 antibody was prepared from mice using the P9 protein as an antigen (Ab Frontier, Korea). The agonist (L-902688) and antagonist (ONO-AE3-208) of EP4 were supplied by Cayman (USA), and restriction enzymes and the T4 DNA ligase were obtained from New England Biolabs (UK). DNA polymerase was purchased from Takara (Japan) and Bioneer (Korea), and oligonucleotide synthesis and DNA sequencing were performed by Bioneer and Cosmogenetech (Korea). HRP-conjugated anti-mouse IgG was obtained from Sigma (USA), and Ni-NTA resin was purchased from Qiagen (Germany). The Superdex200 10/300 GL column was obtained from GE Healthcare (USA). All other consumable reagents were reagent grade.

### 2.2 Construction of EP4 expression vector and expression screening

**Fig. 1:**
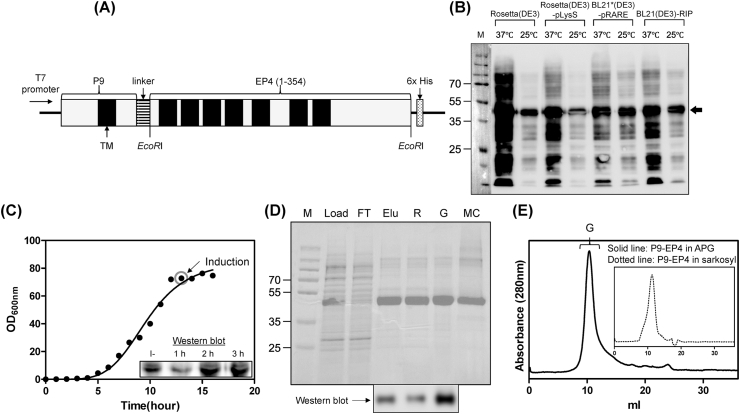
Expression and purification of P9-EP4. (A) Schematic representation of the P9-EP4 expression vector. The coding sequence of human EP4 (amino acids 1–354) was linked to the C-terminus of the P9 protein. The transmembrane regions and the C-terminus His6-tag sequence are indicated as a black box and dotted box, respectively. (B) Expression test of P9-EP4 in various *E. coli* expression hosts. Cells from the culture after induction for 2 h at 37 °C (37 °C), or induction for 3 h at 25 °C (25 °C) were collected and resuspended in an SDS-PAGE sample buffer. The separated proteins were analyzed using western blotting with anti-P9 antibodies. The P9-EP4 band is indicated by an arrow. (C) Culture of BL21(DE3)-RIP cells harboring pP9-EP4 using a fermenter. The expression of P9-EP4 was induced by adding 1 mM IPTG when the OD_600_ nm of the culture media at 37 °C reached 70. Expression level of P9-EP4 in the cells before (−) and after 1, 2, and 3 h of induction was measured using western blotting with anti-P9 antibodies (inserted panel). (D) Purification of P9-EP4. The samples from each purification step were analyzed by SDS-PAGE and proteins were visualized using Coomassie staining. The lanes are described as follows. M: size marker, Load: soluble membrane fraction after solubilization with 1% sarkosyl, FT: flow-through of Ni-NTA chromatography, Elu: eluate from Ni-NTA chromatography, R: reconstituted P9-EP4 with APG, G: purified P9-EP4 stabilized with APG by gel filtration chromatography, and MC: purified EP4 in 0.05% MNG/0.005% CHS. Identical samples used for SDS-PAGE were analyzed using western blotting with anti-6X His-tag antibodies. (E) The elution chromatogram of gel filtration chromatography using Superdex200 10/300 GL of P9-EP4 stabilized with APG. The elution volume that contains P9-EP4 is indicated as G.

The cDNA fragment representing amino acid 1–354 of human EP4 was amplified by PCR reaction with forward primer (5′-TATTTTCAGTCGACGATGGAATTCGAAACCAA CTTCTCCACTCCTCTG-3′) and reverse primer (5′-GTGATGGTGAGAAGCTTCGAATTC CATTGCCTGTAACTCAGTCTCTGC-3′) and ligated at the *EcoR*I site for the pP9 vector (Fig. 1A) [17]. The expression vector of P9-EP4 was transformed into four different *E. coli* strains; Rosetta(DE3), Rosetta(DE3)-pLysS, BL21*(DE3)-pRARE, and BL21(DE3)-RIP. Freshly transformed cells were cultured in 5 ml of Luria-Bertani (LB) medium at 37 °C supplemented with 100 μg/ml ampicillin and 30 μg/ml chloramphenicol until the OD_600_ of the culture reached 0.5–0.6. The expression of P9-EP4 was induced by adding 1 mM IPTG for 2 h at 37 °C, or for 3 h at 25 °C. Cells were harvested by centrifugation at 10,000 g for 20 min, and the expression level of P9-EP4 was visualized by Western blot analysis using the anti-P9 antibody in accordance with standard protocols. The optimum growth condition was selected for scale-up expression.

### 2.3 Expression and purification of P9-EP4

BL21(DE3)-RIP cells harboring pP9-EP4 were grown in 5 ml of LB medium containing 0.1 mg/ml of ampicillin and 0.03 mg/ml of chloramphenicol for 12 h at 37 °C. A 1 ml amount of the pre-culture was inoculated into 50 ml of YTN (yeast extract 1%, bacto tryptone 2%, NaCl 2%) medium containing 0.1 mg/ml of ampicillin and 0.03 mg/ml of chloramphenicol. It was cultured at 37 °C until the OD_600_ nm reached values of 1.0. One-third of the culture was inoculated into 150 ml of YTN medium containing 0.1 mg/ml of ampicillin and 0.03 mg/ml of chloramphenicol and was cultured at 37 °C until the OD_600_ nm reached values of 1.0–2.0. This culture was then inoculated into a fermenter (Marado-PDA, CNS, Korea) containing 3 L of culture medium (K_2_HPO_4_ 0.3%, KH_2_PO_4_ 0.5%, yeast extract 2%, glucose 2%, MgSO_4_·7H_2_O 0.06%, 0.1 mg/ml of ampicillin, and 0.03 mg/ml of chloramphenicol). The cells were grown at 37 °C at pH 6.8 in the presence of ~30% dissolved oxygen. The pH of the culture in the fermenter was maintained at 6.8 by the addition of a small volume of feeding medium (yeast extract 27.4%, (NH_4_)_2_SO_4_ 0.15%, glucose 21.1%, and MgSO_4_·7H_2_O 0.1%). The expression of P9-EP4 was induced by the addition of 1 mM IPTG for 3 h at 25 °C when the OD_600_ nm of the culture was 70. The cells were harvested by centrifugation, and the pellets were stored at −80 °C.

The cell pellets (10 g) were resuspended in 50 mL of buffer A (20 mM HEPES, pH 7.4, 1 mM PMSF, EDTA-free protease inhibitor cocktail) (GenDEPOT, USA), and the cells were lysed using a microfluidizer (M − 110P, Microfluidics, USA). Cell debris was removed from the lysate by centrifugation at 15,000 g for 30 min, and the membrane fraction was recovered from the pellet fraction after centrifugation at 100,000 g for 1 h. The membrane fraction was resuspended with buffer B (20 mM HEPES pH 7.4, 1% sarkosyl, 10 mM β-mercaptoethanol, and 10% glycerol) and the membrane proteins were solubilized by gentle agitation for 1 h at 4 °C. After removing the insoluble materials by centrifugation at 15,000 g for 30 min, the supernatant was loaded onto a Ni-NTA column. After washing the column with 5 mM imidazole in the equilibration buffer, the bound proteins were eluted with 50 mM imidazole. The eluted protein fraction was incubated with amphiphilic polymer APG [17] (mass ratio of P9-EP4/APG equal to 1:4) for 2 h at 4 °C and concentrated with a 10-kDa molecular weight cutoff Amicon Ultra (Millipore, USA) concentrator. The concentrated protein was further purified by size-exclusion chromatography (Superdex200 10/300 GL), which was then pre-equilibrated with buffer C (20 mM HEPES pH 7.4 and 10% glycerol). The purified protein was stored at −80 °C until it was required.

### 2.4 Measurement of the interaction between P9-EP4 and the alpha subunits of G proteins

The alpha subunits of G_s_ and G_i_s were prepared as previously described [19]. G_αs1_, G_αi1_, G_αi2_, or G_αi3_ (10 μg/ml) was immobilized in a high-binding 96-well plate (Corning, USA) (Supplementary information Fig. 1). Each well was blocked with 5% skimmed milk, and various concentrations of purified P9-EP4 in sarkosyl or APG were added to the plate. Subsequently, the plate was successively treated with the anti-P9 antibody, the HRP-conjugated anti-mouse IgG, and 3,3′,5,5′-tetramethylbenzidine. The amount of P9-EP4 bound to the G_α_ was measured using a Synergy H1 (BioTek, USA) microplate reader. The plate was washed extensively at every step, and all procedures were conducted at 25 °C.

### 2.5 Secondary structure analysis and thermostability test of P9-EP4

The circular dichroism (CD) spectra of P9-EP4 were measured in a 1 mm quartz cuvette using a J-715 (JASCO, Japan) spectrometer at 20 °C. The spectra of P9-EP4 in APG (0.063 mg/ml) were recorded in 10 mM sodium phosphate pH 7.4, and the spectra of P9-EP4 in maltose neopentyl glycol/cholesteryl hemisuccinate (MNG/CHS) (0.1 mg/ml) were recorded in 10 mM sodium phosphate pH 7.4, 0.05% MNG, 0.005% CHS. The scanning range was adjusted to 260–190 nm. The spectra were corrected for buffer contributions and were converted to molar ellipticity using a calculated molecular weight of 54401.46 Da. The secondary structure contents of P9-EP4 were predicted using the K2D2 program [20]. The temperature-scanning spectra of P9-EP4 were measured from 10 °C to 100 °C in the 220 nm fixed wavelength.

P9-EP4 in APG, sarkosyl, or MNG/CHS were heated at 80 °C for 30 min; the binding affinity with G_αi1_ was measured by enzyme-linked immunosorbent assay (ELISA) using the same method described in section 2.4.

### 2.6 Analysis of ligand-dependent Trp fluorescence of P9-EP4

The intrinsic fluorescence spectra were measured using a FluoroMate FS-2 (Scinco, Korea) fluorescence spectrometer in a 10 mm quartz cuvette. The excitation wavelength was set to 295 nm, and the emission spectra were recorded in the 310–400 nm range. The ex and em slit widths were set to 10 nm. The P9-EP4 in the APG was adjusted to 2 μM in 50 mM Tris-HCl pH 7.4 and 10% glycerol. The ligands (PGE2, ONO-AE3-208, L-902688) were also adjusted to 2 μM in 50 mM Tris-HCl pH 7.4 and 0.2% ethanol. All spectra were measured after preincubation for 15 min at ~20 °C and were corrected for buffer contributions.

### 2.7 Analysis of antagonist binding by isothermal titration calorimetry (ITC)

ITC experiments were performed using ITC200 instrument (MicroCal Inc.) and the data were analyzed using the program ORIGIN 7.0. The concentration of EP4 protein in the cell were 13 μM, while the syringe contained 200 μM of ONO-AE3-208. The ITC buffer contained 20 mM HEPES pH 7.4, 100 mM NaCl, and up to 1% DMSO. Titrations were carried out at 25 °C, using 13 injections of 2.5 μl each, injected at 150 s intervals. The experimental raw data were corrected for dilution by subtracting the values for buffer alone, and then fit by a one-site binding model. Titration data were fit using a nonlinear least-squares curve-fitting algorithm with three floating variables: stoichiometry, binding constant (K_D_), and change of enthalpy of interaction.

## 3 Results and discussion

### 3.1 Expression and purification of EP4

The P9 expression system in *E. coli* has been successfully applied for the expression of several human GPCR, such as ET_A_, LPA_1_ and LPA_2_, as well as a ligand-gated ion channel (5HT3_A_) [[17], [18], [19],^21^]. This P9 expression system was successively applied for the expression of EP4. The P9 sequence may act as both the leader and anchor sequence that locates the EP4 at the periplasmic membrane of *E. coli*. The optimum conditions for the expression of P9-EP4 were examined in the expression level of P9-EP4 in various *E. coli* expression strains using Western blot analysis. As shown in Fig. 1B, all the tested strains demonstrated similar levels of expression. The expression of P9-EP4 at 25 °C is slightly less than at 37 °C. However, the degradation products of P9-EP4 appeared when the induction temperature was 37 °C. Hence, the mass expression of P9-EP4 was performed using the BL21(DE3)-RIP strain, and the expression of P9-EP4 was induced for 3 h at 25 °C. Typically, ~370 g of wet weight cells was obtained from 3 L of the fermented cell culture, and about ~1 mg of P9-EP4 was expressed at the membrane fraction per 1 g (wet weight) of *E. coli* cells (Fig. 1C). This result indicated that the P9 protein assisted expression system of EP4 in *E. coli* is highly efficient and convenient. This expression system would provide an easy access of purified EP4 for biochemical or biophysical study compared to other expression system of GPCR utilizing mammalian or insect cells.

The P9-EP4 in the membrane fraction was poorly solubilized with non-ionic mild detergents (data not shown). Instead, it was efficiently solubilized with sarkosyl (a strong anionic detergent) and purified using an Ni-NTA affinity column (Fig. 1D). After NTA-affinity chromatography, the purified P9-EP4 was heterogeneously eluted by size-exclusion chromatography (Fig. 1E, dotted line). However, gel-filtration chromatography revealed a mono-dispersed peak (Fig. 1E, solid line) for P9-EP4 stabilized in APG.

### 3.2 Specific interaction of the purified EP4 with the alpha subunits of G proteins

**Fig. 2:**
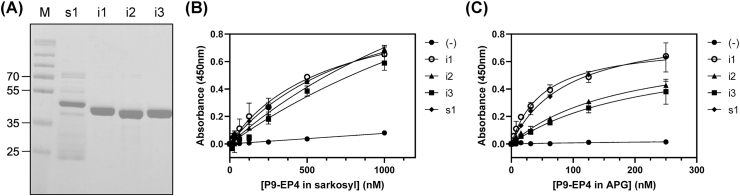
Measurement of the interaction between P9-EP4 and various G protein alpha subunits by ELISA. (A) The purified G_αi1_ (i1), G_αi2_ (i2), G_αi3_ (i3), and G_αs1_ (s1) were analyzed by SDS-PAGE followed by Coomassie staining. (B, C) The concentration-dependent binding of P9-EP4 in sarkosyl (B) or in APG (C) to the immobilized G_α_ proteins. Five to 1000 nM of P9-EP4 was applied to the plate treated with 5% of skimmed milk blocking solution (filled circle), or to the plate immobilized with 10 μg/ml of G_αi1_ (open circle), G_αi2_ (filled triangle), G_αi3_ (filled square), or G_αs1_ (filled diamond), and the amount of bound P9-EP4 was measured using an anti-P9 antibody.

The activation of EP4 transduced the activation of G proteins. It was shown that EP4 was coupled with G_s_ proteins and induced an increase of cAMP at the cellular level [22], suggesting physical interaction with EP4 and G_αs_. Conversely, EP4 was also coupled with G_i_ proteins [14], whose activation resulted in the opposite effects on the cellular concentration of cAMP. However, the relative coupling of EP4 to these G proteins has not yet been investigated. To examine the relative binding of EP4 to the G_s_ or G_i_ proteins, the kinetic parameter of the interaction between the purified P9-EP4 and the G_αs_ or G_αi_ proteins was measured and compared. G_αs1_, G_αi1_, G_αi2_, and G_αi3_ were prepared as previously described [19]. Amount of P9-EP4 bound to the immobilized G_α_ subunits was measured using anti-P9 antibodies. As presented in Fig. 2B and C, P9-EP4 showed substantial binding affinities against the alpha subunits of G proteins. It demonstrated the highest binding affinity (K_D_ = 50 nM) to G_αi1_, and slightly reduced affinities (K_D_ = 88 nM, 175 nM, and 214 nM) to G_αs1_, G_αi2_, and G_αi3_, respectively. These results agree with the previously reported coupling of EP4 to G_s_ and G_i_ proteins. Also, these K_D_ values of P9-EP4 to the alpha subunit of G-proteins is comparable to the affinities of purified GPCRs such as LPA1, LPA2 or endothelin receptors to the alpha subunits of G-proteins [[17], [18], [19]]. It should be noted that the dissociation constants of EP4 to G_αs_ and G_αi_ are similar, suggesting that the relative cellular concentration of G_s_ or G_i_ proteins may be the dominant factor that directs the increase or decrease of cAMP after the activation of EP4.

### 3.3 Conformation and stability of EP4

**Fig. 3:**
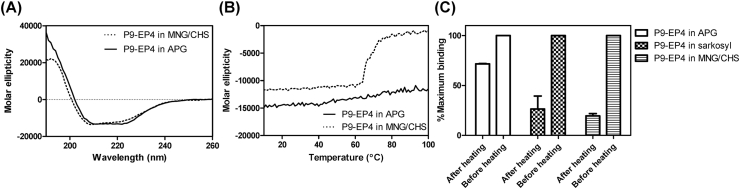
Analysis of secondary structure and thermostability of P9-EP4. (A) The CD spectra of P9-EP4 stabilized with a mixture of MNG and CHS (0.1 mg/ml of P9-EP4 in 10 mM sodium phosphate pH 7.4, 0.05% MNG, and 0.005% CHS; dotted line) or P9-EP4 stabilized with APG (0.063 mg/ml of P9-EP4 in 10 mM sodium phosphate pH 7.4, 0.05% APG: solid line) in the wavelength of 190–260 nm. (B) Temperature-dependent change of the molar ellipticity of P9-EP4 in MNG/CHS (dotted line) or in APG (solid line) at the fixed wavelength of 220 nm. (C) Comparison of the residual binding activity of P9-EP4 to the immobilized G_αi1_ after heat treatment. P9-EP4 in sarkosyl, MNG/CHS or APG were incubated at 80 °C for 30 min, and the amount of bound P9-EP4 was measured using anti-P9 antibodies and compared with the value of P9-EP4 before heat treatment.

The ability of P9-EP4 to interact with G_αs_ and G_αi_ proteins suggests that the purified P9-EP4 are in a natively folded state. To examine the conformation of P9-EP4, the CD spectra of P9-EP4 solubilized with detergent (MNG/CHS or APG) were analyzed. Although P9-EP4 in MNG/CHS or APG showed different size distributions, their CD spectra were almost identical (Fig. 3A). The CD spectrum of P9-EP4 revealed spectra of typical α-helical proteins; the secondary structure content was estimated using the K2D2 program [20]. The helical content of P9-EP4 was predicted as 45% from the CD spectrum. The helical content of P9-EP4 could be estimated from the crystal structure of EP4 [16]. The transmembrane helical regions of EP4 in crystal structure and potential transmembrane helical region of P9 sequence represent 45% of entire sequence (Supplementary information Table 1). Hence, the helical content value of P9-EP4 estimated from the CD spectrum is well matched to the calculated value of helical content. The CD analysis of P9-EP4 along with the specific binding activity of P9-EP4 with G_α_ proteins indicates that the EP4 domain in P9-EP4 has an active conformation.

The method of temperature-dependent denaturation is frequently applied to measure protein stability, which is reflected by the melting temperature. To examine the stability of purified P9-EP4, the temperature-dependent change of molar ellipticity at 220 nm was measured. As presented in Fig. 3B, the P9-EP4 in MNG/CHS revealed a transition at around 65–70 °C, indicating that the protein undergoes thermal denaturation in this temperature range. However, P9-EP4 stabilized in APG showed a slight increase in ellipticity up to 100 °C. Additionally, the P9-EP4 after treatment at 80 °C for 30 min showed comparable binding activity to the G_αi1_ protein, indicating that APG effectively stabilizes P9-EP4. This superior stabilizing activity of APG on EP4 compared with conventional detergent (such as sarkosyl or MNG/CHS) (Fig. 3C) may result from a unique interaction between APG and EP4. APG has more than 20 alkyl chains that can interact with EP4; this multiple interaction may stabilize the APG:P9-EP4 complex even at high temperatures.

### 3.4 Measurement of intrinsic tryptophan fluorescence of EP4 depending on agonist and antagonist interaction

**Fig. 4:**
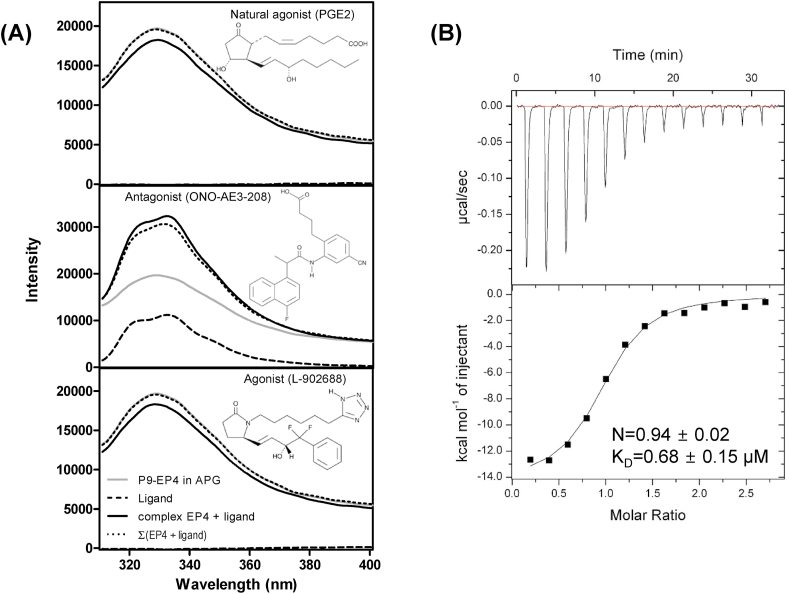
Agonist- and antagonist-binding analysis by the intrinsic fluorescence spectrum of EP4 and ITC.(A) The effects of the endogenous ligand (PGE2), antagonist (ONO-AE3-208), and agonist (L-902688) on the intrinsic fluorescence spectrum of P9-EP4 in APG. The Trp fluorescence spectra of P9-EP4 in APG was measured at 310–400 nm using an excitation wavelength of 295 nm in the absence (gray lines) or presence (solid lines) of chemicals. The spectra of chemicals are indicated as broken lines, and the mathematical summed spectra of chemicals and P9-EP4 are indicated as dotted lines. (B) ITC analysis shows that ONO-AE3-208 binds EP4 with K_D_ = 0.68 ± 0.15 μM at 1:1 M ratio. Raw ITC data (Top panel) and integrated heats of injection (bottom panel) are presented for the titration between EP4 and ONO-AE3-208. In the bottom panel, the experimental data are shown as solid squares and the least squares best-fit curves derived from a simple one-site binding model are shown as a black line.

Various agonistic and antagonistic modulators against EP4 have been identified from cell-based assays and pursued for clinical application. To optimize these modulators, the elucidation of the interaction modes of these compounds with EP4 is required. The transmembrane helical regions of EP4 are assumed to comprise the binding pocket of PGE2. The Arg257 at the 7th transmembrane helix had been important for the binding to PGE2 [16]. However, the extracellular loops are assumed to contribute for the binding of PGE2 as exemplified by the complex structures of S1P_1_ or LPA_1_ with their lipidic ligands [23,24]. To investigate the agonist or antagonist effects on the conformation of EP4, the intrinsic Trp fluorescence of EP4 was examined both in the absence and the presence of agonists and antagonists. P9-EP4 has three Trp residues at the 2nd and 3rd extracellular loops, and one at the 7th transmembrane helical region. A conformational change in P9-EP4 induced by an agonist or an antagonist may alter the microenvironment of these residues and effect a change in the Trp fluorescence spectrum. In the presence of PGE2 and an agonist, the 330 nm peak is substantially quenched. In contrast, the peak is rather de-quenched in the presence of an antagonist (Fig. 4A). The quenching and dequenching of the Trp fluorescence of P9-EP4 in the presence of an agonist and an antagonist, respectively, indicates that the environment of some of these Trp residues are changed to the opposite direction after the agonist/antagonist interaction of P9-EP4. It is assumed that the quenching of Trp fluorescence by agonist interaction induced the Trp residue in the 2nd or 3rd extracellular loop regions, which became more exposed to bulk solvent and enhanced the quenching of the Trp residues; the process is reversed for antagonist interaction. The opposed effect of the agonist or antagonist on the Trp fluorescence of P9-EP4 and the specific interaction of P9-EP4 with G_αs_ and G_αi_ proteins indicates that P9-EP4 has native conformation, which can be exploited for the discovery of novel EP4 modulators. Fig. 4B proves that APG:P9-EP4 complex is native conformation by measuring binding affinity with ONO-AE3-208.

## Author contributions

N.H.K. and Y.G.Y. designed the experiments, analyzed the data and wrote the manuscript. N.H.K., K.-S.K., S.C.S., and E.E.K. performed the experiments.
