# Supplementary material for: Functional expression of human prostaglandin E2 receptor 4 (EP4) in E. coli and characterization of the binding property of EP4 with Gα proteins
Source: Biochem Biophys Rep. 2020 Dec 16;25:100871. doi: 10.1016/j.bbrep.2020.100871 (PMC7749421; doi:10.1016/j.bbrep.2020.100871)
Supplement: Multimedia component 2 [file mmc2.pptx]

## Slide 1
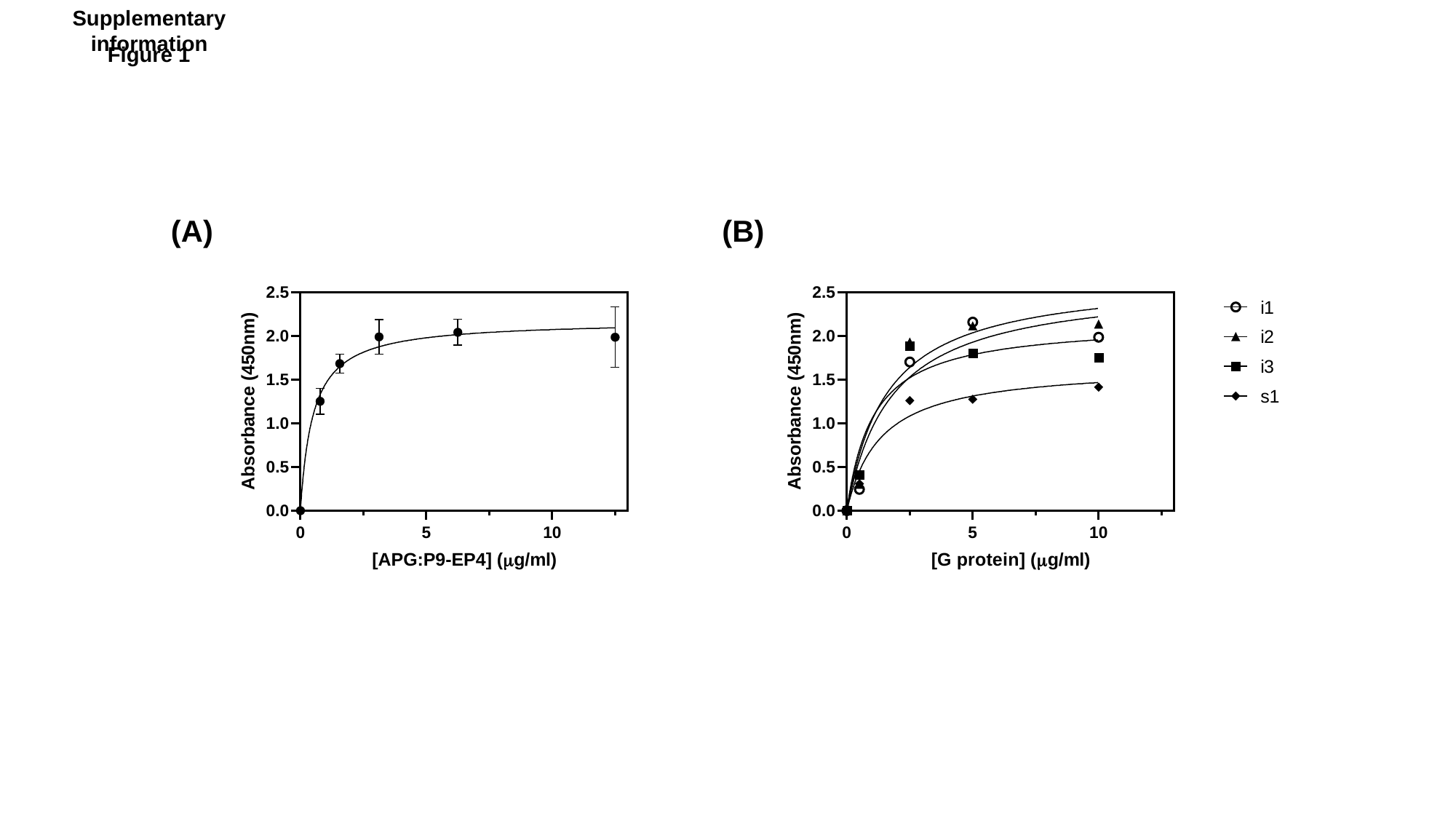

Supplementary information
Figure 1
(A)
(B)

## Slide 2
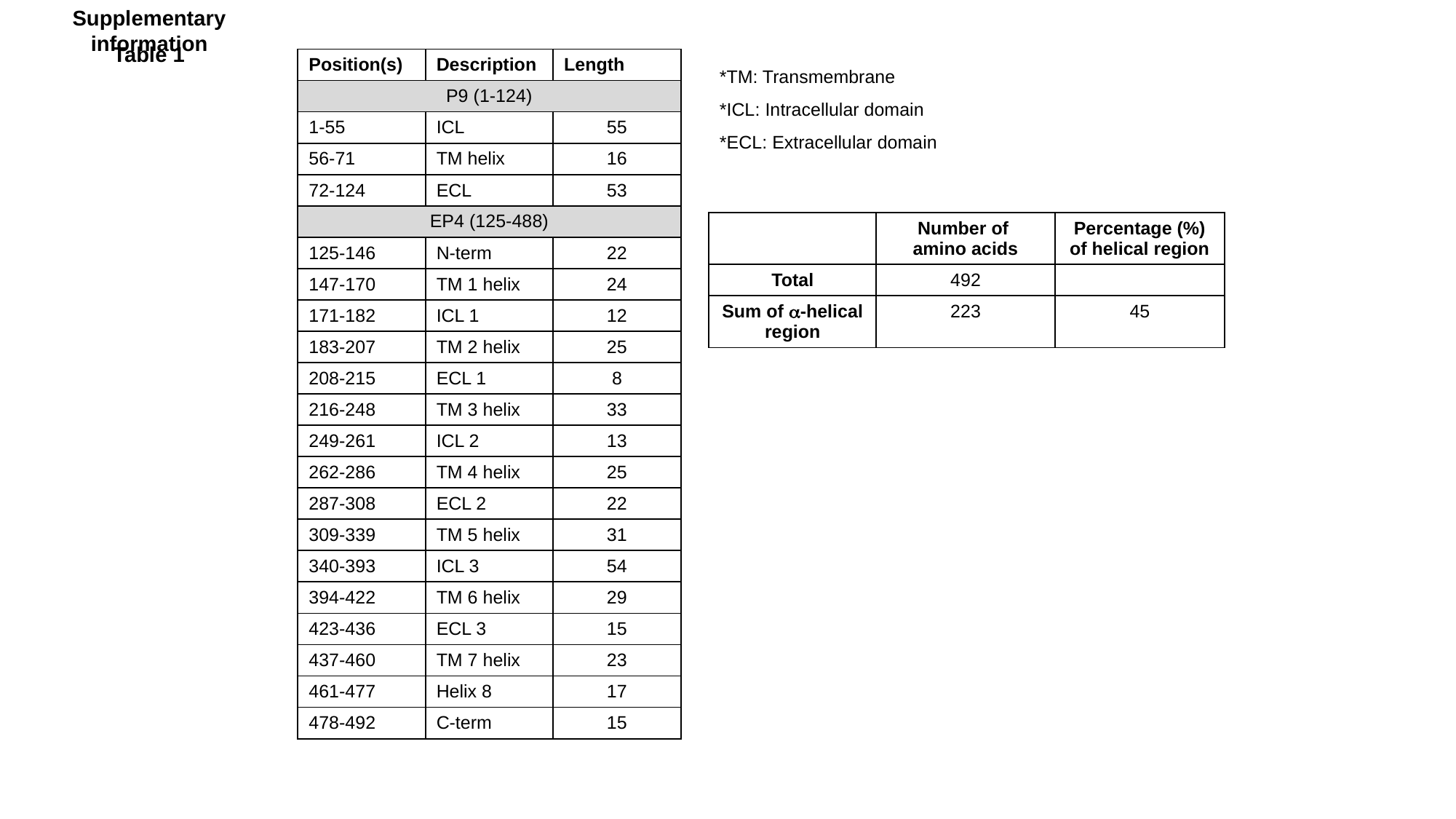

Supplementary information
Table 1
| Position(s) | Description | Length |
| --- | --- | --- |
| P9 (1-124) | | |
| 1-55 | ICL | 55 |
| 56-71 | TM helix | 16 |
| 72-124 | ECL | 53 |
| EP4 (125-488) | | |
| 125-146 | N-term | 22 |
| 147-170 | TM 1 helix | 24 |
| 171-182 | ICL 1 | 12 |
| 183-207 | TM 2 helix | 25 |
| 208-215 | ECL 1 | 8 |
| 216-248 | TM 3 helix | 33 |
| 249-261 | ICL 2 | 13 |
| 262-286 | TM 4 helix | 25 |
| 287-308 | ECL 2 | 22 |
| 309-339 | TM 5 helix | 31 |
| 340-393 | ICL 3 | 54 |
| 394-422 | TM 6 helix | 29 |
| 423-436 | ECL 3 | 15 |
| 437-460 | TM 7 helix | 23 |
| 461-477 | Helix 8 | 17 |
| 478-492 | C-term | 15 |
*TM: Transmembrane
*ICL: Intracellular domain
*ECL: Extracellular domain
| | Number of amino acids | Percentage (%) of helical region |
| --- | --- | --- |
| Total | 492 | |
| Sum of a-helical region | 223 | 45 |
