# Supplementary material for: Functional expression of human prostaglandin E2 receptor 4 (EP4) in E. coli and characterization of the binding property of EP4 with Gα proteins
Source: Biochem Biophys Rep. 2020 Dec 16;25:100871. doi: 10.1016/j.bbrep.2020.100871 (PMC7749421; doi:10.1016/j.bbrep.2020.100871)
Supplement: Multimedia component 3 [file mmc3.docx]

**Functional Expression of Human Prostaglandin E2 Receptor 4 (EP4) in *E. Coli* and Characterization of the Binding Property of EP4 with G_α_ Proteins**

Nam Hyuk Kim^1^, Key-Sun Kim^2^, Sang Chul Shin^3^, Eunice Eunkyeong Kim^3^, and Yeon Gyu Yu^1*^

**Supplementary information**

**Supp. Fig. 1. Immobilization of the alpha subunits of G protein or P9-EP4 on 96-well plate.**

The concentration-dependent binding of P9-EP4 in APG (A) or G proteins (B) to the 96-well plate. Five to 1,000 nM of P9-EP4 or G_αi1_ (open circle), G_αi2_ (filled triangle), G_αi3_ (filled square), or G_αs1_ (filled diamond) were applied in a 96-well plate, and the plate was blocked with skimmed milk (5%). The amounts of bound proteins were measured using an anti-6X His-tag antibodies.

**Supplementary information Table 1. Comparison of secondary structure of P9-EP4 with already known structure of EP4**

The percentage of helix content of P9-EP4 was calculated by dividing total number of amino acid residue (492 aa) by the number of amino acids (223 aa) consisting the 7 transmembrane helical regions and 8^th^ helical region at the cytosolic tail region of EP4 in the crystal structure [16] and the predicted TM helical region in the P9 sequence.
